# Heavy metal contamination in unrecorded rakia from Kosovo and its public health implications

**DOI:** 10.1038/s41598-025-03338-4

**Published:** 2025-05-31

**Authors:** Teuta Muhollari, Sándor Szűcs, Martin McKee, Róza Ádány, Zsófi Sajtos, Edina Baranyai, László Pál

**Affiliations:** 1 Department of Public Health and Epidemiology, Faculty of Medicine, University of Debrecen, P.O. Box 400, Debrecen, 4002 Hungary; 2 Department of Health Services Research and Policy, London School of Hygiene and Tropical Medicine, London, United Kingdom; 3 HUN-REN-UD Public Health Research Group, Department of Public Health and Epidemiology, Faculty of Medicine, University of Debrecen, Debrecen, Hungary; 4 Department of Inorganic and Analytical Chemistry, Atomic Spectroscopy Laboratory, University of Debrecen, Debrecen, Hungary

**Keywords:** Unrecorded alcohol, Lead, Home-made rakia, Kosovo, Balkan countries, Health risk assessment, Risk factors, Public health

## Abstract

Excessive alcohol consumption poses a challenge to public health across Europe, but especially in the Balkan region, where alcohol from unrecorded sources, particularly home-distilled rakia, is widely consumed. Our previous research identified elevated lead and copper levels in rakia from these sources collected in Albania. However, due to its disputed political status and absence from many international health surveys, data on the composition of home-made rakia from Kosovo remains scarce. To address this knowledge gap, we report an analysis of 30 rakia samples from unrecorded sources collected in Kosovo, measuring 24 elements, including toxic metals, using inductively coupled plasma optical emission spectrometry. The ethanol concentration of the samples was determined by gas chromatography/mass spectrometry. In addition, we have applied the target hazard quotient and the margin of exposure (MOE) methods to assess the associated health risks. We find that the threshold limits of copper, iron, nickel, and lead have been exceeded in 96.7%, 3.3%, 3.3% and 26.7% of rakia samples, respectively. The health risk assessment showed that the MOE value of lead for both average and heavy drinkers was less than 100 when consuming unrecorded rakia, indicating potential for adverse health effects. Along with previous research, these findings highlight the need for targeted public health research in the Balkan region to better understand the impact of unrecorded alcohol on disease burden and mortality and how to develop policies that strengthen enforcement of trading standards.

**Supplementary Information:**

The online version contains supplementary material available at 10.1038/s41598-025-03338-4.

## Introduction

In 2021, the Global Burden of Disease project estimated that high alcohol use was the leading contributor, among its second-level risk factors, to the disease burden and to deaths among those aged 15–49 in the countries of central Europe (accounting for 11.3% of disease burden and 15.6% of deaths in this age group), overtaking tobacco, which had occupied that position in 1990^1^. It was ranked fourth among men aged 50–69^1^.

While much of this burden can be attributed to consumption of ethanol, there is growing concern about products from unregulated, and therefore unrecorded sources^2^. These sources include smuggling and cross-border shopping, distillation in private homes or small-scale enterprises, and industrial alcohols not intended for consumption^3^. It has been estimated that alcohol from these sources account for 16.7%, 14.5%, 13.7%, 10.4%, and 6.7% of total per capita ethanol intake in North Macedonia, Bosnia and Herzegovina, Albania, Serbia and Montenegro, respectively^4^.

This concern stems from findings that these unrecorded spirits can contain considerably higher concentrations not just of ethanol but also other harmful substances, including methanol, ethyl-carbamate and heavy metals than their counterparts from recorded sources^2,5–11^. Consequently, it has been suggested that consumption of such beverages can increase the risk of alcohol-related diseases, especially among heavy drinkers^5–7,12^.

One home-distilled product, rakia, is especially important in these countries^3,6,13^. There are few data on its composition but our previous analyses of home-produced Albanian rakia detected lead (Pb) and copper (Cu) at concentrations exceeding threshold values proposed by the Alcohol Measures for Public Health Research Alliance (AMPHORA) project^6^. Although the levels that we detected were not high enough to cause acute toxicity, chronic exposure to low concentrations of Pb is considered a risk factor for kidney diseases, neurological disorders, and anaemia^14–16^. Consequently, we cannot definitively exclude the possibility that consumption of unrecorded rakia could have adverse effects for health^6^.

In a follow up study, we have analysed samples of rakia obtained in the Republic of Kosovo (RK). RK was previously a province of Serbia but is now de facto independent but, as it is not recognised as such by all members of the Security Council, is not a member of the United Nations. It has an estimated population of 1.6 million, 90% of whom are ethnic Albanians^17^. Its disputed political status means that participation in international health surveys is limited, so there are few data on the amounts and types of alcohol consumed. However, given its shared history, it is likely that consumption patterns are similar to those in other Balkan countries, including frequent consumption of rakia. This raises the possibility that Kosovar drinkers may also be exposed to elevated levels of heavy metals but, so far, we are unaware of any data on the chemical composition of home-made rakia in the RK.

This study extends our previous research by determining the concentration of several elements among them toxic metals including cadmium (Cd), chromium (Cr), cobalt (Co), Cu, nickel (Ni), and Pb in samples of rakia from unrecorded sources collected in the RK. Our main objective is to ascertain the proportion of rakia samples in which levels of metals detected exceed the toxicological threshold values established by the AMPHORA project for unrecorded alcohols and to estimate the resulting health risk posed to consumers. This is the first study to analyse rakia from unrecorded sources in the RK.

## Materials and methods

### Sample collection

We described our approach to sampling in our previous paper^6^. Briefly, we purchased thirty samples of rakia from small-scale and sustenance producers. None of the rakia samples had the tax stamp that appears on officially recorded products. We then transferred each sample into glass bottles with identification numbers to avoid any potential mismatches and stored them at 4 °C until we could conduct gas chromatographic mass spectrometric (GC/MS) and inductively coupled plasma optical emission spectrometric (ICP-OES) analyses. We recorded where each sample was purchased, the raw materials reported to be used in production, ethanol content as reported by the producers, price per litre, and the date of sampling.

### Gas chromatographic mass spectrometric analysis

#### Materials

Acetonitrile and ethanol were supplied by Merck (Darmstadt, Germany) and used as internal and qualitative standards for the analysis of rakia samples, respectively. All reagents employed were of high-performance liquid chromatography grade.

#### Determination of ethanol concentration in rakia samples

We have described in detail how we determined ethanol concentration in our previous article^6^. Briefly, we analysed the rakia samples using a Hewlett-Packard (HP) GC/MS system (Palo Alto, CA, USA) consisting of a HP 6890 GC, a HP 5973 mass selective detector (MSD) and an Agilent 7683 automatic liquid sampler (Agilent Technologies, Palo Alto, CA, USA). A Hewlett-Packard Free Fatty Acid Phase (length: 50 m, internal diameter: 0.2 mm, film thickness: 0.33 μm) cross-linked capillary column (Hewlett-Packard, Palo Alto, CA, USA) was used for the separation of ethanol using helium as carrier gas at 3.0 bar constant pressure.

### Inductively coupled plasma optical emission spectrometric analysis

#### Materials

Nitric acid and mono-element spectroscopic standards were obtained from ESLab (Debrecen, Hungary), while the multi-element spectroscopic standard solution and hydrogen peroxide were purchased from Merck (Darmstadt, Germany). Ultrapure water was prepared using a Synergy UV water purification system (Merck Millipore, Darmstadt, Germany). All reagents used were of spectroscopic or reagent grade.

#### Determination of element levels in rakia samples

The concentrations of 24 elements were determined using an Agilent ICP-OES system (model 5100 VDV, Agilent Technologies, Santa Clara, USA) as previously described in detail^6^. We used an autosampler (Agilent SPS4), a Meinhard nebuliser and a double-pass spray chamber to introduce the samples. Argon gas was used for plasma supply. Standard arsenic (As) and tin (Sn) solutions were prepared from mono-element spectroscopic standards with a concentration of 1000 mg/litre. In addition, multi-element standards containing all other elements at a concentration of 1000 mg/litre were used to prepare calibration solutions for silver (Ag), aluminium (Al), boron (B), bismuth (Bi), calcium (Ca), Cd, Co, Cr, Cu, iron (Fe), gallium (Ga), indium (In), potassium (K), lithium (Li), magnesium (Mg), manganese (Mn), sodium (Na), Ni, Pb, strontium (Sr), thallium (Tl), and zinc (Zn). A five-point calibration curve was used for the quantitative analysis. Three parallel measurements were carried out for each sample. To validate the ICP-OES method, blank samples were used to check the purity of water and glassware used. The influence of ethanol concentration on the detectability of heavy metals was determined by preparing solutions containing ethanol (Merck, Darmstadt, Germany) at concentrations of 0.0, 5.0, 10.0, 25.0, and 40.0% spiked with the multi-element standards. The final concentration of the elements was 0.1 mg/L. The accuracy of the measurements was above 95%. The operating conditions of the ICP-OES are given in Supplement 1.

### Comparison of metal concentrations in unrecorded rakia with threshold values

The metal concentrations measured in the rakia samples were compared with the limits proposed by the AMPHORA project^18^. The levels of metals were expressed in milligrams per litre of pure alcohol (p.a.) to allow comparison with the results of previous research.

### Statistical analysis of ethanol levels in rakia samples

**Fig. 1:**
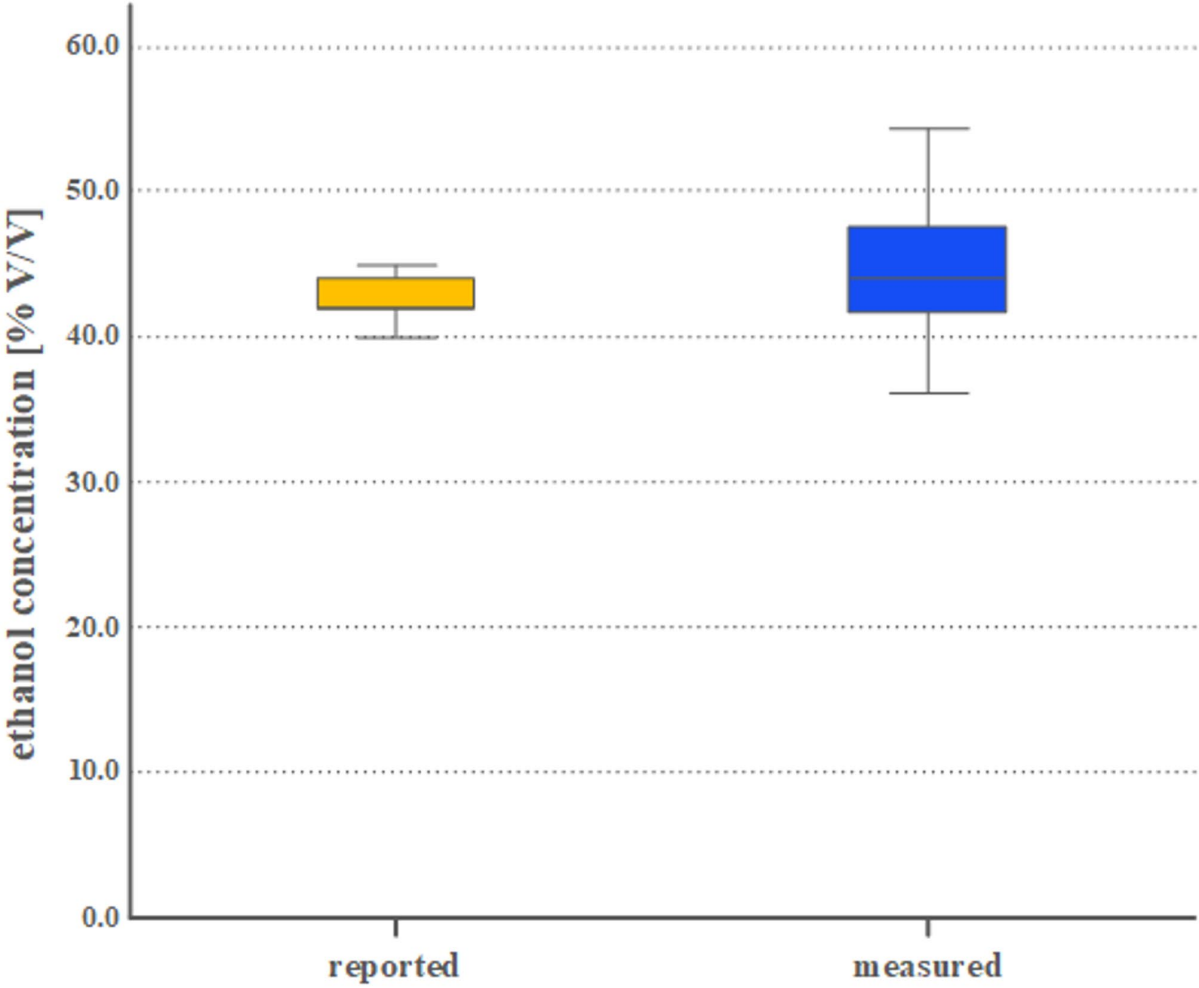
Differences between reported and measured ethanol concentrations of rakia samples. The concentration of ethanol [% volume/volume] in the unrecorded Kosovar rakia samples was reported by the sample provider (boxplot coloured yellow) and also determined by gas chromatographic and mass spectrometric measurements (boxplot coloured blue). There was no statistically significant difference between the two categories. The p value was larger than 0.05.

The ethanol levels reported by the providers of rakia samples (reported) were compared to those determined in the GC/MS analyses (measured). The distribution of the data was evaluated for normality using the Shapiro–Wilk test, which indicated that the data were not normally distributed. Therefore, we used the Wilcoxon signed-rank test to evaluate the mean differences between the reported and measured ethanol concentrations. We conducted the statistical analysis using IBM’s SPSS software version 25.0 (IBM Inc., Armonk, New York, USA). We considered a p-value of less than 0.05 to be statistically significant. The median ethanol concentration, along with its interquartile range (IQR) and 1.5 times the IQR (shown as whiskers), are illustrated in Fig. 1.

### Estimation of health risks

The toxicological thresholds proposed by the AMPHORA project are based on extrapolation of drinking water quality guidelines and standards of the WHO and European Union (EU), respectively^18^. Assuming an average daily per capita consumption of alcohol ten times lower than that of drinking water, the AMPHORA limits were calculated by multiplying the WHO and EU guidelines by 10. Drinking water standards established by the WHO and EU are based on a number of toxicological and epidemiological considerations and indicate the concentrations of chemical contaminants, including heavy metals, in drinking water that do not pose a significant health risk to consumers, even in the case of lifetime exposure^19^. Therefore, it is unlikely that the consumption of unrecorded rakia containing concentrations of heavy metals below the AMPHORA limits can result in a health risk. Consequently, to determine the health risk associated with consuming unrecorded rakia, we estimated the daily intake of those metals that exceeded the AMPHORA threshold value in at least one of the samples collected. Accordingly, Cu, Fe, Ni and Pb were selected. In those instances where their concentration was below the limit of detection (LOD), the LOD values of the selected metals (see Table 1) were divided by two and the entries indicated in the database as “< LOD” were replaced with the result of the calculation. Next, the median and the 99th percentile values of the concentration of the selected metals (see Table 2) were calculated. Then these data were used to estimate the target hazard quotient (THQ) values if a reference dose had previously been reported for the selected metals^5^. As a result, the median and the 99th percentile values of the concentration of Cu, Fe and Ni were applied in the following formula:

$$EDI = \frac{MDI \times MCS}{{BW}}$$

where, the EDI is the estimated daily intake, the MDI is the mass of the daily ethanol intake in g/day, MCS is the median and the 99th percentile values of the concentration of Cu, Fe and Ni in mg/g of p.a. and BW is the average body weight of 73.9 kg for both sexes^5^. The health risk estimation considered two scenarios: “average drinkers” and "heavy drinkers”. Although data on the volume of unrecorded alcohol intake in the RK are not available, we assumed that it would be similar to the levels reported for both sexes aged 15 years and above in neighbouring Balkan countries, such as Albania (0.7 L of p.a./capita/year), Montenegro (0.7 L of p.a./capita/year), North Macedonia (0.8 L of p.a./capita/year), and Serbia (0.8 L of p.a./capita/year)^4^. Averaging these volumes, the intake of ethanol from homemade rakia in the RK was estimated to be 0.75 L of p.a./capita/year, assuming that all unrecorded alcohol was consumed in that form. Subsequently, the weight of unrecorded alcohol consumed annually was calculated by multiplying 0.75 L by the density of ethanol (0.789 g/cm^3^), resulting in a quantity of 591.8 g/year. This is equivalent to the consumption of 1.62 g of ethanol per day. This value was used in the scenario termed “average drinkers”. The second scenario, termed "heavy drinkers”, was defined as the consumption of 60 g of ethanol per day in unrecorded rakia^4^. Subsequently, THQ values were calculated^5^. The THQ is a method used to estimate the non-carcinogenic risk related to chronic exposure to chemicals such as metals^5^. It is calculated as the ratio of the oral dose of a metal to its reference level^5^. A ratio exceeding 1.0 is interpreted as an elevated health risk, while values less than 1.0 are considered negligible^5^. To calculate the THQ values, the following factors were taken into account: the EDI, the exposure frequency (EF, 365 days/year), the exposure duration (ED, years), the average exposure time (AET, 365 days/year × ED) and the oral reference dose of the particular metals (RfD, mg/kg/day, see Table 1)^5,20^. The ED was defined as the number of years that an average Kosovar person can be expected to live at the age of 15 (65.4 years for both sexes)^21^. The following equation was used for the health risk estimation:

$$THQ = \frac{MCS \times MDI \times EF \times ED}{{RfD \times BW \times AET}}$$

**Table 1:** Oral reference dose, lower bound of the benchmark dose confidence interval, and limit of detection values of metals selected for health risk assessment.

Heavy metal	Reference dose [mg/body weight kg/day]	Limit of detection [mg/litre]
Cu	0.04	1.0 × 10^–4^
Fe	0.7	2.0 × 10^–4^
Ni	0.011	5.0 × 10^–4^
Pb*	0.0015^a^	2.2 × 10^–3^
	0.00063^b^	

*The reference dose for lead have not been reported therefore, the lower bound of the benchmark dose confidence interval, expressed in mg/body weight kg/day, was used in the health risk assessment for lead.

^a^Lower bound of the benchmark dose confidence interval for cardiovascular effects in adults (BMDL_01_).

^b^Lower bound of the benchmark dose confidence interval for nephrotoxicity in adults (BMDL_10_).

**Table 2:** Median and 99th percentile values of the concentration of metals in Kosovar rakia.

Heavy metal	Median [mg/g of p.a.]*	99th percentile [mg/g of p.a.]
Cu	0.01	0.05
Fe	1.5 × 10^–4^	2.1 × 10^–3^
Ni	3.2 × 10^–7^	3.8 × 10^–3^
Pb	8.3 × 10^–5^	3.4 × 10^–3^

*The concentration of the metals is expressed in mg/g of pure alcohol (p.a.).

Since a RfD value for Pb has not been reported, the margin of exposure (MOE) approach was applied to estimate the health risk associated with drinking Kosovar rakia containing this heavy metal^22^. As recommended by the European Food Safety Authority (EFSA), this is the preferred method for assessing the risk to human health from the presence of Pb in food^16^. The MOE can be defined as the ratio of the lower bound of the benchmark dose confidence interval (BMDL) of a substance to the estimated intake of the same substance^16,22^. As the MOE value highly depends on the health endpoints for which the BMDL was determined, we considered those outcomes that were found to be the most critical in adults, including cardiovascular effects and nephrotoxicity using BMDL values of 0.0015 (BMDL_01_) and 0.00063 (BMDL_10_) mg/body weight kg/day, respectively^16^. MOE values less than 100 are considered as a public health concern for substances not classified as genotoxic or carcinogenic, including Pb^22^. The MOE values were calculated as follows:

$$MOE=\frac{BMDLx}{EDI}$$

where the BMDL_x_ is the BMDL value reported for a specific health endpoint.

## Results

Table 3 reports the raw materials used to produce the 30 samples: 20 (66.7%), 2 (6.7%), 1 (3.3%), 1 (3.3%), 3 (10%), and 1 (3.3%) were produced from fermented grapes, pears, mulberries, apples, plums, and quince, respectively. In two samples (6.7%), this was not provided. Five rakia samples were produced between 2013 and 2015, while 24 were distilled later, between 2017 and 2022. This information was not available for one sample (Table 3). The ethanol content was reported on labels of 90% (n = 27) of the samples, varying between 38.0% and 60.0% vol/vol [% v/v] (Table 3). Finally, Table 3 reports the price of home-made rakia, between 2.5 and 10.0 euros/litre.

**Table 3:** Characteristics of rakia samples.

Sample number	Place of sample collection	Raw material	Year of distillation	Reported ethanol concentration (% v/v)^a^	Measured ethanol concentration (% v/v)^b^	Difference between measured and reported ethanol concentration (% v/v)	Price/litre (euros)
Sample 1	Producer’s home	Grape	2019	Unknown	42.3	Not applicable^c^	3.5
Sample 2	Flea market	Pear	2020	42.0	40.1	− 1.9^d^	2.5
Sample 3	Flea market	Mulberry and honey	2020	42.0	48.6	6.6	2.5
Sample 4	Flea market	Apple	2020	43.0	46.4	3.4	2.5
Sample 5	Flea market	Grape	2020	40.0	43.3	3.3	2.5
Sample 6	Flea market	Plum	2020	38.0	42.2	4.2	2.5
Sample 7	Flea market	Quince	2020	45.0	41.8	− 3.2	4.0
Sample 8	Producer’s home	Grape	2017	41.0	40.3	− 0.7	2.5
Sample 9	Producer’s home	Grape	2022	42.0	42.2	0.2	5.0
Sample 10	Producer’s home	Grape	2022	44.0	44.2	0.2	3.0
Sample 11	Producer’s home	Grape	2018	42.0	42.0	0	4.0
Sample 12	Producer’s home	Grape	2022	43.0	44.9	1.9	5.0
Sample 13	Producer’s home	Grape	2022	40.0	44.0	4.0	5.0
Sample 14	Producer’s home	Grape	2022	48.0	47.6	− 0.4	7.0
Sample 15	Producer’s home	Grape	2015	43.0	51.8	8.8	5.0
Sample 16	Producer’s home	Grape	2022	42.0	41.0	− 1.0	3.0
Sample 17	Producer’s home	Grape	2022	44.0	44.0	0	5.0
Sample 18	Producer’s home	Unknown	2017	Unknown	48.6	Not applicable	Unknown
Sample 19	Producer’s home	Plum	2021	40.0	50.3	10.3	10.0
Sample 20	Producer’s home	Unknown	Unknown	Unknown	48.1	Not applicable	Unknown
Sample 21	Producer’s home	Grape	2018	43.0	47.5	4.5	3.0
Sample 22	Producer’s home	Grape	2018	42.0	45.0	3.0	3.0
Sample 23	Producer’s home	Grape	2021	44.0	48.8	4.8	3.5
Sample 24	Producer’s home	Grape	2021	42.0	47.4	5.4	4.0
Sample 25	Producer’s home	Grape	2015	60.0	65.1	5.1	4.0
Sample 26	Producer’s home	Grape	2018	42.0	43.4	1.4	5.0
Sample 27	Producer’s home	Plum	2013	45.0	42.6	− 2.4	6.0
Sample 28	Producer’s home	Pear	2015	42.0	37.9	− 4.1	8.0
Sample 29	Producer’s home	Grape	2015	45.0	41.1	− 3.9	3.0
Sample 30	Producer’s home	Grape	2021	60.0	54.3	− 5.7	3.5

^a^Information on ethanol concentration were reported by the provider of the rakia sample.

^b^The concentration of ethanol was determined by gas chromatographic mass spectrometric analysis.

^c^The difference between the measured and reported ethanol concentration cannot be calculated.

^d^The negative difference between the measured and reported ethanol concentrations indicates that the reported values were higher than those obtained from our measurements.

The GC/MS analyses estimated ethanol levels from 37.9 to 65.1% v/v and there was no significant difference between the reported (mean: 43.6% v/v, IQR 42.0–44.0% v/v) and measured ethanol levels in the samples (mean: 45.0% v/v, IQR 42.0–47.0% v/v), (Fig. 1, *p* > 0.05).

The results of the ICP-OES analyses are shown in Table 4. Al was detected in all rakia samples (n = 30) at concentrations ranging from 0.034 to 0.560 mg/l of p.a., which are below the AMPHORA threshold of 2.0 mg/l of p.a. The level of Cu exceeded the recommended limit of 2.0 mg/l of p.a. in 96.7% (n = 29) of the samples, with concentrations varying between 1.033 and 36.536 mg/l of p.a. Only a single rakia sample contained Fe at a concentration that surpassed the threshold value of 2.0 mg/l of p.a., with levels ranging from 0.049 to 0.965 mg/l of p.a. in the remaining 29 samples. Mn was found in all samples at levels varying from 0.004 to 0.077 mg/l of p.a., all less than the threshold of 0.5 mg/l of p.a. Although Ni was detected in four samples only, one had a concentration 20-fold higher than the AMPHORA limit of 0.2 mg/l of p.a. Pb was detected in 76.7% (n = 23) of the samples, the threshold of 0.2 mg/l of p.a. was exceeded in 26.7% of the samples (n = 8). All samples had Zn concentrations below the threshold of 5.0 mg/l of p.a., ranging between 0.028 and 2.898 mg/l of p.a.

**Table 4:** Concentration of metals detected in Kosovar rakia samples.

AMPHORA^b^ threshold values of metals	Concentration of detected metals^a^						
	Al [mg/l]	Cu [mg/l]	Fe [mg/l]	Mn [mg/l]	Ni [mg/l]	Pb [mg/l]	Zn [mg/l]
	2.0	2.0	2.0	0.5	0.2	0.2	5.0
Sample 1	0.068	**18.221**^**c**^	0.205	0.014	< LOD^d^	**0.204**	1.631
Sample 2	0.530	**3.031**	0.206	0.030	0.024	< LOD	0.150
Sample 3	0.125	**7.682**	0.224	0.074	< LOD	0.071	0.049
Sample 4	0.040	**33.524**	0.170	0.004	< LOD	0.152	0.246
Sample 5	0.038	**21.962**	0.108	0.005	< LOD	0.181	0.180
Sample 6	0.087	**25.837**	**2.035**	0.019	< LOD	**0.233**	0.289
Sample 7	0.097	**8.458**	0.250	0.077	0.028	0.096	0.187
Sample 8	0.170	**21.978**	0.156	0.020	< LOD	0.130	2.898
Sample 9	0.035	**3.989**	0.073	0.009	< LOD	< LOD	0.052
Sample 10	0.065	**6.311**	0.065	0.018	< LOD	< LOD	0.244
Sample 11	0.121	**9.394**	0.102	0.010	< LOD	0.101	0.190
Sample 12	0.059	**8.333**	0.166	0.004	**4.231**	**0.206**	0.053
Sample 13	0.038	**15.939**	0.092	0.005	< LOD	0.060	0.332
Sample 14	0.048	**12.444**	0.098	0.004	< LOD	**0.282**	0.038
Sample 15	0.040	**10.763**	0.086	0.004	< LOD	0.028	0.046
Sample 16	0.085	**21.057**	0.119	0.010	< LOD	0.050	0.190
Sample 17	0.042	**15.298**	0.088	0.009	< LOD	**0.628**	0.050
Sample 18	0.112	**11.480**	0.178	0.012	< LOD	0.038	0.066
Sample 19	0.097	**2.663**	0.049	0.004	< LOD	< LOD	0.028
Sample 20	0.043	**6.161**	0.109	0.004	< LOD	0.030	0.042
Sample 21	0.246	**19.236**	0.119	0.004	< LOD	**1.276**	0.080
Sample 22	0.081	**20.763**	0.095	0.004	< LOD	**1.321**	0.071
Sample 23	0.034	**36.536**	0.051	0.008	< LOD	**3.238**	0.324
Sample 24	0.136	**6.842**	0.149	0.076	< LOD	< LOD	0.346
Sample 25	0.069	**6.291**	0.124	0.061	< LOD	< LOD	0.295
Sample 26	0.038	**11.570**	0.965	0.005	< LOD	0.111	0.124
Sample 27	0.560	**5.158**	0.218	0.028	< LOD	0.048	0.263
Sample 28	0.139	1.033	0.318	0.021	< LOD	0.017	0.063
Sample 29	0.089	**22.942**	0.162	0.015	< LOD	< LOD	0.263
Sample 30	0.101	**24.032**	0.104	0.007	0.018	0.056	0.862

^a^Concentrations of metals were determined by inductively coupled plasma optical emission spectrometric analysis (ICP-OES) and expressed in mg/liter (mg/l) of pure alcohol.

^b^AMPHORA: Alcohol Measures for Public Health Research Alliance.

^c^Bold values indicate rakia samples containing metals at levels above the AMPHORA threshold values. The concentrations of other elements detected in rakia samples are presented in the Supplement 3.

^d^< LOD: The concentration of metals was below the limit of detection (LOD) of the ICP-OES.

The results of our health risk assessment are presented in Tables 5 and 6. The THQ values were below 1.0 for Cu, Fe, and Ni in both scenarios (Table 5). The MOE values for average drinkers consuming unrecorded rakia containing Pb at the 99th percentile concentration were below 100 for both cardiovascular effects and nephrotoxicity. For both health outcomes, the MOEs for heavy drinkers exposed to Pb from unrecorded rakia were less than 100 at the median and 99th percentile concentrations (Table 6).

**Table 5:** Target hazard quotient values for an average and heavy Kosovar drinker consuming unrecorded rakia.

	Average drinkers^a^		Heavy drinkers^b^	
	THQ^c^	THQ^d^	THQ^c^	THQ^d^
Cu	8.0 × 10^–3^	2.4 × 10^–2^	2.9 × 10^–1^	0.9
Fe	4.8 × 10^–6^	6.8 × 10^–5^	1.8 × 10^–4^	2.5 × 10^–3^
Ni	6.3 × 10^–7^	7.6 × 10^–3^	2.3 × 10^–5^	2.8 × 10^–1^

Target hazard quotients (THQ) values were calculated using the reference doses of the selected heavy metals.

^a^Average drinkers were defined as those with a consumption of 1.62 g of ethanol per day from unrecorded rakia.

^b^Heavy drinkers were defined as those with a consumption of 60 g of ethanol per day from unrecorded rakia.

^c^The median concentrations of the selected metals in unrecorded rakia were used to calculate the THQ.

^d^The 99th percentile concentrations of the selected metals in unrecorded rakia were used to calculate the THQ.

**Table 6:** Margin of exposure values for average and heavy Kosovar drinkers consuming unrecorded rakia containing lead.

Health outcome	Average drinkers^a^		Heavy drinkers^b^	
	MOE^c^	MOE^d^	MOE^c^	MOE^d^
Cardiovascular effects	824.1	20.0	22.3	0.5
Nephrotoxicity	346.1	8.4	9.3	0.2

Margin of exposure (MOE) values were calculated using the lower confidence limit of the benchmark dose (BMDL) determined for cardiovascular effects (BMDL_01_: 0.0015 mg/ body weight kg/day) and nephrotoxicity (BMDL_10_: 0.00063 mg/ body weight kg/day).

^a^Average drinkers were defined as those with a consumption of 1.62 g of ethanol per day from unrecorded rakia.

^b^Heavy drinkers were defined as those with a consumption of 60 g of ethanol per day from unrecorded rakia.

^c^The median concentration of Pb in unrecorded rakia was used to calculate the MOE.

^d^The 99th percentile concentration of Pb in unrecorded rakia was used to calculate the MOE.

## Discussion

We have extended our previous study by determining the concentration of 24 elements in 30 samples collected in the RK, thereby helping to close a gap in our knowledge of concentrations of toxic metals in Kosovar rakia and any associated health risks^6,23–25^. We found that 97% (n = 29) of the samples analysed had ethanol concentrations greater than 40% v/v, the typical alcohol strength of rakia^26,27^. We also found no significant difference between the levels of ethanol reported on labels and what was measured in unrecorded spirit samples. This is in contrast with our previous findings, which found a discrepancy between the measured (mean: 46.7% v/v, IQR 43.4–52.1% v/v) and reported (mean: 18.9% v/v, IQR 17.0–20.0% v/v) alcohol strength of unrecorded Albanian rakia^6^. This could be due to differences in if, where, and how Albanian producers have their samples analysed but this cannot be determined without further enquiry. We also found, perhaps unsurprisingly given the strong cultural ties and the similarities in production, that the ethanol concentrations determined by GC/MS analysis in the two countries were similar (Kosovar samples: mean: 45.0% v/v, IQR 42.0–47.0% v/v) and those in our previous investigation (Albanian rakia, mean: 46.7% v/v, IQR 43.4–52.1% v/v)^6^.

As in our earlier Albanian study, Cu and Pb were identified as the most concerning heavy metals in the RK samples^6^. The proportions of samples where these exceeded the AMPHORA thresholds were similar (Cu: 90%, Pb: 33%) and Kosovar (Cu: 97%, Pb: 27%)^6,18^. Similarly, mean levels of Cu and Pb in the Kosovar rakia were nearly equivalent to those in the samples collected in Albania (Cu: 13.1 ± 9.09 mg/l of p.a., Pb: 0.2 ± 0.32 mg/l of p.a.) and Kosovo (Cu: 13.9 ± 9.21 mg/l of p.a., Pb: 0.3 ± 0.65 mg/l of p.a.)^6^. This was also consistent with a study by Torović et al. of unrecorded Serbian fruit spirits, which had an average Cu concentration of 8.18 ± 8.37 mg/l of p.a.^23^.

These findings suggest that home-made production of rakia in the Balkans frequently involves substandard distillation equipment, which may contaminate spirits with heavy metals^6,23^.

To assess the health risk associated with the consumption of unrecorded rakia, we calculated the THQ and MOE values. The THQ values for Cu, Fe and Ni were below 1.0, indicating that the health risk from drinking unrecorded rakia containing these heavy metals is negligible for both average and heavy drinkers. However, for cardiovascular effects and nephrotoxicity, the MOE values were less than 100, showing an increased health risk for both "average and heavy drinkers" consuming rakia containing Pb at the median (8.3 × 10^–5^ mg/g of p.a.) and 99th percentile concentrations (3.4 × 10^–3^ mg/g of p.a.).

This supports previous research finding an increased health risk for consumers of unrecorded spirits due to the presence of Pb at concentrations exceeding threshold values^6^. Given the relatively low price of home-made rakia (mean ± standard deviation: 4.19 ± 1.81 euros/litre), the actual health risk may be even greater than estimated, as heavy drinkers are likely to consume larger quantities of ethanol from unrecorded rakia than assumed in our risk assessment^5,28^. However, further studies are needed to investigate this hypothesis.

The strengths and limitations of our study should also be considered. Our study is the first to determine the concentrations of 24 elements, among them many heavy metals, in unrecorded Kosovar rakia. We have shown that a large proportion of rakia samples contained Cu and Pb at levels above the AMPHORA threshold values. In addition, the health risk assessment indicated that heavy drinkers of unrecorded rakia may be at increased risk due to Pb exposure from consumption. However, our study is limited by the small number of rakia samples analysed. Also, our risk assessment assumes that the volume of alcohol consumed in the RK is comparable to that in the neighbouring countries, which may not necessarily be true. The ethanol intake used in our study for “heavy drinkers” may be lower than their actual alcohol consumption, which could mean that we underestimated the health risks associated with exposure to Cu and Pb.

Consistent with previous research, our study confirms that heavy metals, including Cu, Fe, Ni and Pb, can be present in unrecorded rakia at concentrations above the AMPHORA thresholds and may pose a public health concern. Of these, Pb was identified as the most significant, with the potential to increase the health risk to heavy drinkers.

Our findings have implications for research and policy. First, there are many alcohol products manufactured in this region that are drunk without anyone really knowing what they contain. While this study has focused on heavy metals, previous research has found high levels of various long-chain alcohols in other unrecorded spirits in this region^7,11,29^. Patterns of risk factors and disease in this region are relatively understudied, leaving many questions unanswered, such as the long-term effects of consuming these products. There is some evidence, for example, that chronic lead exposure can affect the heart^30^ and it is only relatively recently that a dietary cause of another disease occurring in this region, Balkan nephropathy, was elucidated^31^. There is also an obvious need for strengthening capacity for inspection and regulation of these products although, given their place in culture and how, historically, they have been produced and distributed^32^, this will not be easy.

## Electronic supplementary material

Below is the link to the electronic supplementary material.

Supplementary Material 1

## Data Availability

All data on the concentration of elements and measurement conditions were provided in the manuscript and supplementary files.
