# Supplementary material for: Heavy metal contamination in unrecorded rakia from Kosovo and its public health implications
Source: Sci Rep. 2025 May 31;15:19150. doi: 10.1038/s41598-025-03338-4 (PMC12126508; doi:10.1038/s41598-025-03338-4)
Supplement: Supplementary file 1 — Supplementary Material 1 [file 41598_2025_3338_MOESM1_ESM.docx]

**Supplement 1**

**Heavy metal contamination in unrecorded rakia from Kosovo and its public health implications**

Teuta Muhollari^a^, Sándor Szűcs^a^, Martin McKee^b^, Róza Ádány^a,c^, Zsófi Sajtos^d^, Edina Baranyai^d^ and László Pál^a*^

^a^Department of Public Health and Epidemiology, Faculty of Medicine, University of Debrecen, Debrecen, Hungary; ^b^Department of Health Services Research and Policy, London School of Hygiene and Tropical Medicine, London, United Kingdom; ^c^HUN-REN-UD Public Health Research Group, Department of Public Health and Epidemiology, Faculty of Medicine, University of Debrecen, Hungary; ^d^Department of Inorganic and Analytical Chemistry, Atomic Spectroscopy Laboratory, University of Debrecen, Debrecen, Hungary

E-mail addresses: teutamuhollari@gmail.com (T. Muhollari); szucs.sandor@med.unideb.hu (S. Szűcs); martin.mckee@lshtm.ac.uk (M. McKee); adany.roza@med.unideb.hu (R. Ádány); sajtos.zsofi@science.unideb.hu (Zs. Sajtos); baranyai.edina@science.unideb.hu (E. Baranyai); pal.laszlo@med.unideb.hu (L. Pál)

*Please address all correspondence to: Dr. László Pál, Department of Public Health and Epidemiology, Faculty of Medicine, University of Debrecen, H-4012 Debrecen, P.O. Box 9, Hungary. (T) 3652512765 (F) 3652417267 (E) pal.laszlo@med.unideb.hu

**Supplementary table 1.** Information on unrecorded Kosovar rakia samples

| sample number | Country | location of sample collection | location of sample production | vessel material^a^ | storage place^b^ | temperature^c^ [˚C] | exposure to light | colour |
| --- | --- | --- | --- | --- | --- | --- | --- | --- |
| Sample 1 | Kosovo | Gjakovë | Gjakovë | plastic | shelf | 20 | No | Yellowish |
| Sample 2 | Kosovo | Ferizaj | Ferizaj | plastic | shelf | 10 | Yes | Yellowish |
| Sample 3 | Kosovo | Ferizaj | Ferizaj | plastic | shelf | 10 | Yes | Yellowish |
| Sample 4 | Kosovo | Ferizaj | Ferizaj | plastic | shelf | 10 | Yes | -^d^ |
| Sample 5 | Kosovo | Ferizaj | Ferizaj | plastic | shelf | 10 | Yes | - |
| Sample 6 | Kosovo | Ferizaj | Ferizaj | plastic | shelf | 10 | Yes | - |
| Sample 7 | Kosovo | Ferizaj | Ferizaj | plastic | shelf | 10 | Yes | - |
| Sample 8 | Kosovo | Rahovec | Rahovec | plastic | cellar | 13 | No | Colorless |
| Sample 9 | Kosovo | Rahovec | Rahovec | plastic | cellar | 17 | No | Yellowish |
| Sample 10 | Kosovo | Rahovec | Rahovec | plastic | cellar | 14 | No | Colorless |
| Sample 11 | Kosovo | Rahovec | Rahovec | plastic | cellar | 10 | No | Colorless |
| Sample 12 | Kosovo | Rahovec | Rahovec | plastic | shelf | 17 | Yes | Colorless |
| Sample 13 | Kosovo | Rahovec | Rahovec | plastic | cellar | 10 | No | Colorless |
| Sample 14 | Kosovo | Rahovec | Rahovec | plastic | cellar | 12 | No | Colorless |
| Sample 15 | Kosovo | Rahovec | Rahovec | plastic | cellar | 17 | No | Colorless |
| Sample 16 | Kosovo | Rahovec | Rahovec | plastic | cellar | 18 | No | Yellowish |
| Sample 17 | Kosovo | Rahovec | Rahovec | plastic | cellar | 17 | No | Colorless |
| Sample 18 | Kosovo | Pejë | Vitomericë | glass | shelf | 27 | No | Colorless |
| Sample 19 | Kosovo | Pejë | Pejë | glass | refrigerator | 3 | No | Yellowish |
| Sample 20 | Kosovo | Pejë | Vitomericë | glass | shelf | 27 | No | Colorless |
| Sample 21 | Kosovo | Rahovec | Rahovec | plastic | cellar | 15 | No | Colorless |
| Sample 22 | Kosovo | Rahovec | Rahovec | plastic | cellar | 15 | No | Colorless |
| Sample 23 | Kosovo | Rahovec | Rahovec | plastic | shelf | 21 | Yes | Colorless |
| Sample 24 | Kosovo | Rahovec | Rahovec | wood barrel | cellar | 12 | No | Brownish |
| Sample 25 | Kosovo | Rahovec | Rahovec | wood barrel | storage room | 20 | No | Brownish |
| Sample 26 | Kosovo | Rahovec | Rahovec | plastic | refrigerator | 4 | No | Colorless |
| Sample 27 | Kosovo | Rahovec | Rahovec | glass | shelf | 21 | Yes | Yellowish |
| Sample 28 | Kosovo | Rahovec | Rahovec | plastic | shelf | 21 | Yes | Yellowish |
| Sample 29 | Kosovo | Rahovec | Rahovec | stainless steel (inox) tank | cellar | 6.6 | No | Colorless |
| Sample 30 | Kosovo | Rahovec | Rahovec | plastic | cellar | 6 | No | Colorless |

^a^Vessel material describes the matter of container in which fruit spirits were kept before sample collection.

^b^Storage place describes the space where fruit spirits were kept before sample collection.

^c^Temperature of the storage place.

^d^Information on the colour of the sample was not collected at the time of the sampling.

**Supplementary table 2.** ICP-OES conditions I

| common conditions | |
| --- | --- |
| Replicates | 3 |
| Pump speed | 15 rpm |
| Uptake time | 15 sec |
| Rinse time | 30 sec |
| Read time | 5 sec |
| RF power | 1.20 kW |
| Stabilization time | 15 sec |
| Nebulizer flow | 0.70 L min^.1^ |
| Plasma flow | 12.0 L min^.1^ |
| Aux flow | 1.0 L min^.1^ |
| Viewing height | 8 mm |
| Viewing mode | axial |

**Supplementary table 3.** ICP-OES conditions II

| element | wavelength  (nm) |
| --- | --- |
| Ag | 328.068 |
| Al | 396.152 |
| As | 188.980 |
| B | 249.772 |
| Ba | 455.403 |
| Bi | 223.061 |
| Ca | 422.673 |
| Cd | 422.673 |
| Cr | 267.716 |
| Cu | 324.754 |
| Fe | 238.204 |
| Ga | 417.204 |
| In | 303.396 |
| K | 766.491 |
| Li | 670.783 |
| Mg | 279.553 |
| Mn | 257.610 |
| Na | 589.592 |
| Ni | 216.555 |
| Pb | 220.353 |
| Sn | 189.925 |
| Sr | 407.771 |
| Tl | 351.923 |
| Zn | 213.857 |

**Supplementary table 4.** Concentrations of other elements in Kosovar rakia samples using inductively coupled plasma optical emission spectrometric analysis

|  |  | | | | | | |  | |  | |  | |  | |  | |  | |  |
| --- | --- | --- | --- | --- | --- | --- | --- | --- | --- | --- | --- | --- | --- | --- | --- | --- | --- | --- | --- | --- |
|  | **As**  **[mg/l]^a^** | **B**  **[mg/l]** | **Ba**  **[mg/l]** | **Bi**  **[mg/l]** | **Ca**  **[mg/l]** | **Cd**  **[mg/l]** | **Co**  **[mg/l]** | | **Cr**  **[mg/l]** | **K**  **[mg/l]** | **Li**  **[mg/l]** | | **Mg**  **[mg/l]** | | **Na**  **[mg/l]** | | **Sn**  **[mg/l]** | | **Sr**  **[mg/l]** | |
| AMPHORA^b^  threshold values of metals | **0.1** | **5.0** | **0.01** | **0.5** | **no limit** | **0.2** | **1.0** | | **0.5** | **no limit** | **no limit** | | **no limit** | | **no limit** | | **1.0** | | **no limit** | |
| sample 1 | <LOD^c^ | 0.029 | 0.008 | <LOD | 4.510 | <LOD | <LOD | | 0.003 | 9.407 | 0.008 | | 1.271 | | 9.651 | | <LOD | | 0.009 | |
| sample 2 | <LOD | 0.569 | 0.008 | <LOD | 9.899 | <LOD | <LOD | | 0.003 | 499.105 | <LOD | | 21.805 | | 2.988 | | <LOD | | 0.005 | |
| sample 3 | <LOD | 0.062 | 0.019 | <LOD | 20.057 | <LOD | <LOD | | <LOD | 19.763 | <LOD | | 1.353 | | 1.643 | | 0.188 | | 0.021 | |
| sample 4 | <LOD | <LOD | 0.003 | <LOD | 2.978 | <LOD | <LOD | | 0.003 | 1.063 | <LOD | | 0.469 | | 1.044 | | <LOD | | 0.004 | |
| sample 5 | <LOD | 0.005 | 0.017 | <LOD | 3.196 | <LOD | <LOD | | <LOD | 0.755 | <LOD | | 0.443 | | 0.818 | | <LOD | | 0.005 | |
| sample 6 | <LOD | 0.001 | 0.003 | <LOD | 54.260 | <LOD | <LOD | | 0.003 | 0.813 | <LOD | | 0.814 | | 0.939 | | <LOD | | 0.019 | |
| sample 7 | <LOD | 0.259 | 0.022 | <LOD | 71.746 | <LOD | <LOD | | 0.003 | 326.036 | <LOD | | 15.559 | | 2.604 | | <LOD | | 0.029 | |
| sample 8 | <LOD | 0.031 | 0.008 | <LOD | 5.974 | <LOD | <LOD | | <LOD | 1.313 | <LOD | | 0.669 | | 3.644 | | <LOD | | 0.020 | |
| sample 9 | <LOD | 0.010 | 0.003 | <LOD | 4.146 | <LOD | <LOD | | <LOD | 3.249 | <LOD | | 0.781 | | 1.285 | | <LOD | | 0.005 | |
| sample 10 | <LOD | 0.001 | 0.003 | <LOD | 7.293 | <LOD | <LOD | | <LOD | 0.554 | <LOD | | 0.506 | | 1.159 | | <LOD | | 0.009 | |
| sample 11 | <LOD | 0.010 | 0.003 | <LOD | 3.552 | <LOD | <LOD | | <LOD | 0.417 | <LOD | | 0.294 | | 0.834 | | <LOD | | 0.005 | |
| sample 12 | <LOD | 0.023 | 0.003 | <LOD | 1.732 | <LOD | <LOD | | <LOD | 0.595 | <LOD | | 0.173 | | 1.390 | | <LOD | | 0.004 | |
| sample 13 | <LOD | 0.005 | 0.003 | <LOD | 2.927 | <LOD | <LOD | | <LOD | 1.070 | <LOD | | 0.267 | | 5.069 | | <LOD | | 0.009 | |
| sample 14 | <LOD | 0.005 | 0.003 | <LOD | 1.793 | <LOD | <LOD | | 0.003 | 0.338 | <LOD | | 0.151 | | 0.778 | | <LOD | | 0.004 | |
| sample 15 | <LOD | 0.012 | 0.003 | <LOD | 1.571 | <LOD | <LOD | | <LOD | 0.373 | <LOD | | 0.243 | | 1.607 | | <LOD | | 0.004 | |
| sample 16 | <LOD | 0.093 | 0.003 | <LOD | 6.365 | <LOD | <LOD | | <LOD | 12.695 | <LOD | | 1.024 | | 3.089 | | <LOD | | 0.005 | |
| sample 17 | <LOD | <LOD | 0.003 | <LOD | 2.440 | <LOD | <LOD | | 0.003 | 0.180 | <LOD | | 0.277 | | 0.973 | | <LOD | | 0.009 | |
| sample 18 | <LOD | 0.046 | 0.003 | <LOD | 1.971 | <LOD | <LOD | | 0.003 | 0.471 | <LOD | | 0.238 | | 2.359 | | <LOD | | 0.004 | |
| sample 19 | <LOD | 0.084 | 0.003 | <LOD | 1.327 | <LOD | <LOD | | 0.003 | 1.414 | 0.007 | | 0.349 | | 2.744 | | <LOD | | 0.004 | |
| sample 20 | <LOD | 0.001 | 0.003 | <LOD | 4.577 | <LOD | <LOD | | <LOD | 0.551 | <LOD | | 0.236 | | 2.313 | | <LOD | | 0.004 | |
| sample 21 | <LOD | 0.026 | 0.003 | <LOD | 2.260 | <LOD | <LOD | | <LOD | 0.962 | <LOD | | 0.197 | | 918.759 | | <LOD | | 0.004 | |
| sample 22 | <LOD | 0.023 | 0.012 | <LOD | 3.653 | <LOD | <LOD | | <LOD | 1.122 | <LOD | | 0.417 | | 743.090 | | <LOD | | 0.009 | |
| sample 23 | <LOD | 0.013 | 0.007 | <LOD | 4.737 | <LOD | <LOD | | <LOD | 0.686 | 0.007 | | 1.684 | | 1.152 | | <LOD | | 0.012 | |
| sample 24 | <LOD | 0.047 | 0.011 | <LOD | 20.324 | <LOD | <LOD | | 0.003 | 7.167 | <LOD | | 4.725 | | 23.710 | | <LOD | | 0.055 | |
| sample 25 | <LOD | 0.080 | 0.005 | <LOD | 1.407 | <LOD | <LOD | | <LOD | 4.410 | <LOD | | 0.334 | | 17.727 | | <LOD | | 0.003 | |
| sample 26 | <LOD | <LOD | 0.003 | <LOD | 1.594 | <LOD | <LOD | | <LOD | 0.371 | <LOD | | 0.188 | | 0.609 | | <LOD | | 0.005 | |
| sample 27 | <LOD | 0.034 | 0.008 | <LOD | 11.699 | <LOD | <LOD | | 0.008 | 19.585 | <LOD | | 3.074 | | 74.625 | | <LOD | | 0.014 | |
| sample 28 | <LOD | 0.064 | 0.009 | <LOD | 3.836 | <LOD | <LOD | | 0.004 | 9.712 | <LOD | | 1.424 | | 43.953 | | <LOD | | 0.005 | |
| sample 29 | <LOD | 0.006 | 0.008 | <LOD | 5.177 | <LOD | <LOD | | 0.003 | 2.226 | <LOD | | 1.162 | | 1.616 | | <LOD | | 0.015 | |
| sample 30 | <LOD | 0.004 | 0.006 | <LOD | 1.263 | <LOD | <LOD | | 0.002 | 0.389 | <LOD | | 0.169 | | 1.293 | | <LOD | | 0.004 | |

^a^Concentrations of metals were determined by inductively coupled plasma optical emission spectrometric analysis (ICP-OES) and expressed in mg/liter (mg/l) of pure alcohol.

^b^AMPHORA: Alcohol Measures for Public Health Research Alliance (see the manuscript for more information on threshold values of heavy metals in unrecorded spirits)

^c^<LOD: The concentration of metals was below the limit of detection (LOD) of the ICP-OES.
